# Simultaneous Multislice Brain MRI T1 Mapping with Improved Low-Rank Modeling

**DOI:** 10.3390/tomography7040047

**Published:** 2021-10-07

**Authors:** Sugil Kim, Suhyung Park

**Affiliations:** 1 Siemens Healthineers Korea Ltd., Seoul 03737, Korea; sugil.kim@siemens-healthineers.com; 2 Department of Brain and Cognitive Engineering, Korea University, Seoul 02841, Korea; 3 Department of Computer Engineering, Chonnam National University, Gwangju 61186, Korea; 4 Department of ICT Convergence System Engineering, Chonnam National University, Gwangju 61186, Korea

**Keywords:** magnetic resonance imaging, simultaneous multislice, low rank, null space, T1 mapping

## Abstract

To accelerate data acquisition speed in magnetic resonance imaging (MRI), multiple slices are simultaneously acquired using multiband pulses. Simultaneous multislice (SMS) imaging typically unfolds slice aliasing from the acquired collapsed slices. In this study, we extended the SMS framework to accelerated MR parameter quantification such as T1 mapping. Assuming that the slice-specific null space and signal subspace are invariant along the parameter dimension, we formulated the SMS framework as a constrained optimization problem under a joint reconstruction framework such that the noise and signal subspaces are used for slice separation and recovery, respectively. The proposed method was validated on 3T MR human brain scans. We successfully demonstrated that the proposed method outperforms competing methods in suppressing aliasing artifacts and noise at high SMS accelerations, thus leading to accurate T1 maps.

## 1. Introduction

In magnetic resonance imaging (MRI), quantitative parameter mapping, which includes voxelwise delineation of tissue-specific relaxation times, has been widely used in characterizing inherent tissue properties and evaluating various pathological diseases, thereby providing valuable insight into disease processes. These voxels serve as imaging markers for clinical applications such as acute stroke, epilepsy, and multiple sclerosis [1,2,3,4]. As parameter mapping in MRI requires that several repeated measurements with varying imaging parameters, it may result in prohibitively long imaging times, thus compromising imaging efficiency and possibly limiting a range of clinical applications.

In accelerated MR parameter mapping, simultaneous multislice (SMS) imaging has gained attention owing to its ability to provide the signal-to-noise ratio (SNR) benefit of volumetric signal averaging by simultaneously exciting multiple imaging slices [5,6,7]. Parallel imaging techniques have been used to unfold the overlapped slices by solving an inverse problem consisting of multicoil linear system equations [8,9,10,11,12]. To obtain better noise suppression and artifact mitigation over conventional data acquisition, a controlled aliasing (CAIPI) technique was introduced to SMS acquisition by applying phase-modulated RF excitation to best utilize the multicoil encoding power that maximizes the sensitivity variation between neighboring slices [12,13]. CAIPI acquisition, coupled with parallel imaging, provides higher SNR efficiency due to the improved conditioning of the matrix inversion. To flexibly control intra- and interslice artifacts, split slice-GRAPPA (SP-SG) imposes a slice leakage constraint by effectively passing signals in a slice of interest while nulling the leakage terms of the other slices [14,15,16]. However, as simultaneously acquired slices are close to each other, the neighboring slices may share spatial correlation to a certain extent, thus limiting the use of a single reconstruction kernel.

To enable fast MR parameter mapping for a large number of slices, an extended SMS Hankel subspace learning (HSL) framework, which is the generalization of SMS-HSL [17] that exploits the Hankel structured matrix property in *k*-space, was developed in this study. For slice unfolding, the null subspace of the acquired SMS data is combined with the signal subspace to recover the signal within a single slice. To this end, the parameter dimension was considered for dynamic SMS imaging by combining the Hankel matrices from different contrast images to yield a larger but more redundant low-rank matrix model that consists of signal and noise subspaces. In vivo experiments were performed using SMS single-shot EPI data sets with multiple inversion recovery times for T1 mapping to validate the capability of the extended SMS-HSL algorithm at high SMS factors.

## 2. Materials and Methods

### 2.1. Introduction to SMS-HSL

An SMS signal can be represented as a linear combination of multiple slices:

(1) $$y=\sum_{s}^{N_{s}}x_{s}+n$$

where $y\in {\mathbb{C}}^{N_{x}\times N_{y}\times N_{c}}$ is the zero-filled measured signal, $y\in {\mathbb{C}}^{N_{x}\times N_{y}\times N_{c}}$ is the *s*th slice signal, and $n\in {\mathbb{C}}^{N_{x}\times N_{y}\times N_{c}}$ is the measurements noise. To make the best of redundant information from intra- and intercorrelations in *k*-space, each slice signal can be rewritten as a Hankel matrix form [18]:

(2) $$H\left(y\right)=\sum_{s}^{N_{s}}H\left(x_{s}\right)+N$$

where $H\left(\cdot \right):\;{\mathbb{C}}^{N_{x}\times N_{y}\times N_{c}}\to {\mathbb{C}}^{\left(N_{x}-r+1\right)\left(N_{y}-r+1\right)\times r^{2}N_{c}}$ the Hankel operator that maps the k-space data into a Hankel-structured matrix by vectorizing r×r rectangular k-space data across all coils inside the window followed by stacking the vectors row-wise as the sliding window shifts to generate different rows. In multiband excitation, the above signal can be decomposed on the basis of a dual slice model into 1. the slice of interest and 2. its complementary slices:

(3) $$H\left(y\right)=H\left(x_{s}\right)+H\left(x_{s}^{c}\right)+N$$

where $x_{s}^{c}$ is the complementary slice signal and is defined as the linear superposition of all simultaneously excited slices excluding the slice of interest. The Hankel matrices in Equation (3) are then projected onto the different subspaces spanned by the complementary null space $N_{s}^{c}$.

(4) $$H\left(y\right)N_{s}^{c}=H\left(x_{s}\right)N_{s}^{c}+H\left(x_{s}^{c}\right)N_{s}^{c}\approx H\left(x_{s}\right)N_{s}^{c}$$

Accordingly, the aliasing separation in the slice direction is performed by solving the following least squares problem:

(5) $$x_{s}=\underset{x_{s}}{\min}\left\|(H\left(y\right)-H\left(x_{s}\right)\right)N_{s}^{c}\|{}_{F}^{2}$$

Additionally, as the multicoil images become redundant by sharing identical information across the coil dimension, the Hankel structured data matrix becomes highly rank-deficient, thus imposing a low-rank prior under the SMS reconstruction framework.

(6) $$E\left(x_{s}\right)=\underset{x_{s}}{\min}\frac{1}{2}\left\|(H\left(y\right)-H\left(x_{s}\right)\right)N_{s}^{c}\|{}_{F}^{2}+\lambda_{\ell }\|H\left(x_{s}\right){\|}_{*}$$

where $\|\cdot {\|}_{*}$ is the nuclear norm defined as the sum of the singular values of the matrix.

### 2.2. Extension to Parameter Dimension

**Figure 1:**
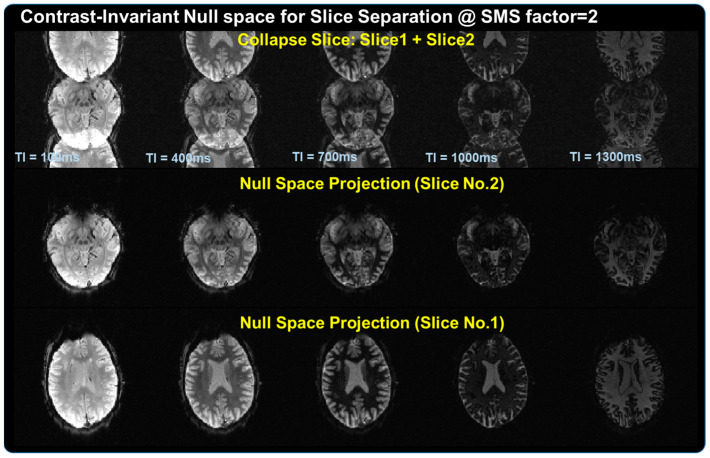
An illustration of the use of null space for slice separation. This work hypothesizes that the null space is invariant along the parameter dimension. The null space projection effectively filters out a slice of no interest while passing through a slice of interest regardless of contrast changes.

**Figure 2:**
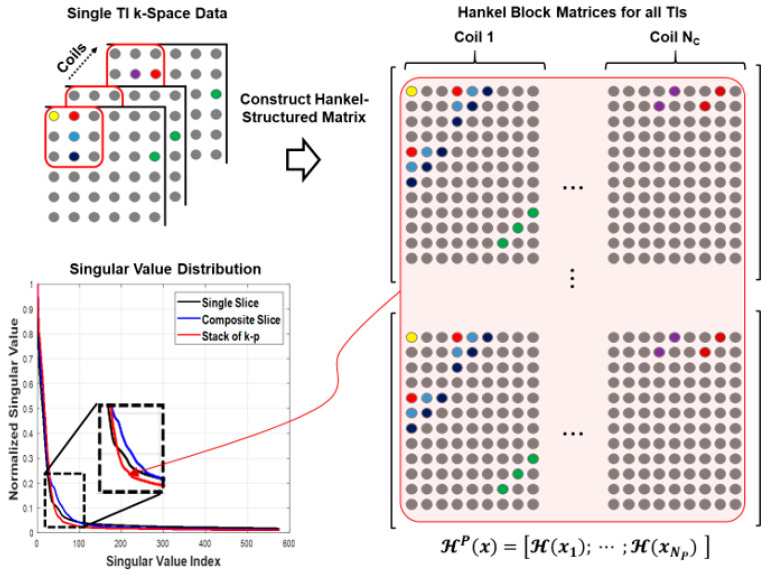
Utility of low rank in Hankel structured matrix. The larger Hankel matrices, which combine all parameter *k*-space data, are more rank-deficient than single and composite slices, which implies that the matrix completion scheme is more suitable to the application of MR parameter mapping.

The simplest approach to applying SMS-HSL to parameter mapping is to independently reconstruct each parameter image. As the null space is observed to be contrast-invariant along the parameter dimension, the null space is applied to the individual parameter images, thus leading to signal nulling in a slice of no interest while passing a slice of interest for all TIs (Figure 1). To make better low-rank characteristics in multicontrast imaging, the larger Hankel matrices $H^{P}\left(\cdot \right):\;{\mathbb{C}}^{N_{x}\times N_{y}\times N_{c}\times N_{p}}\to {\mathbb{C}}^{\left(N_{x}-r+1\right)\left(N_{y}-r+1\right)N_{p}\times r^{2}N_{c}}$, formulated as:

(7) $$H^{P}\left(x_{s}\right)=\left[H\left(x_{s,1}\right);\;H\left(x_{s,2}\right);\cdots ;\;H\left(x_{s,N_{p}}\right)\right]$$

can be constructed by concatenating the Np different contrast Hankel-structured matrices in the parameter dimension. The Hankel-structed low- rank properties can be observed by applying singular value decomposition (SVD) to a single slice, linearly combined composite SMS slice, and multicontrast block matrices, respectively. Figure 2 shows the corresponding singular value distributions. The block matrix shows that singular values drop quite rapidly compared to different matrices. This implies that the conversion of the individual parameter matrices into a larger multicontrast matrix increases the rank deficiency, thus making the matrix completion concept of SMS-HSL more suitable to MR parameter mapping in SMS imaging. This implies that the conversion of the individual parameter matrices into a larger multiparameter matrix increases the rank deficiency, thus making the matrix completion concept of SMS-HSL more suitable to MR parameter mapping in SMS imaging. In addition, the larger Hankel matrices potentially have a larger number of null space vectors as a result of the increased low rank of the Hankel structured matrix. This suggests that the null space projection term in Equation (6) improves the performance while unfolding the collapsed slices into individual slices by utilizing the increased redundancy of the null space. As a result, we computed the null space vectors using larger Hankel matrices obtained by:

(1) concatenating the individual parameter Hankel structured matrices;
(2) applying SVD to block Hankel matrix;
(3) taking the right singular vectors corresponding to the low singular values.

Similar to the SMS-HSL based reconstruction formula of Equation (7), the proposed reconstruction problem can be formulated as:

(8) $$E\left(x_{s}\right)=\underset{x_{s}}{\min}\overset{N_{p}}{\underset{p=1}{\sum }}\frac{1}{2}\|\left(H\left(y_{p}\right)-H\left(x_{s,p}\right)\right)N_{s}^{c}\|{}_{F}^{2}+\lambda_{\ell }\|H^{P}\left(x_{s}\right){\|}_{*}$$

The first term measures how well the collapsed slices are separated into individual slices, and the second regularization term provides a powerful signal recovery constraint for each slice by converting the slice separation problem into a single slice signal recovery problem. For the sake of brevity, Equation (8) is represented by concatenating the Hankel structured matrix for each contrast:

(9) $$E\left(x_{s}\right)=\underset{x_{s}}{\min}\|\left(H^{P}\left(y\right)-H^{P}\left(x_{s}\right)\right)N_{s}^{c}\|{}_{F}^{2}+\lambda_{\ell }\|H^{P}\left(x_{s}\right){\|}_{*}$$

where $H^{P}\left(y_{p}\right)\;\mathrm{and}\;H^{P}\left(x_{s}\right)$ are defined as:

(10) $$H^{P}\left(y\right)=\left[H\left(y_{1}\right);\;H\left(y_{2}\right);\cdots ;\;H\left(y_{N_{P}}\right)\right],\;H^{P}\left(x_{s}\right)=\left[H\left(x_{s,1}\right);\;H\left(x_{s,2}\right);\cdots ;\;H\left(x_{s,N_{p}}\right)\right]$$

As the proposed reconstruction framework of Equation (8) is highly similar to the SMS-HSL cost function, the same optimization algorithm can be used to minimize both terms. In this study, the low-rank matrix fitting approach (LMaFit) was used under the framework of an alternating direction method (ADM) [9] to solve the aforementioned formulation.

### 2.3. Optimization Algorithm

Increasing the number of parameter dimensions makes Equation (8) computationally intractable. Instead, we employ the LMaFit approach, which does not use SVD, to substantially mitigate the computational complexity. The algorithm factorizes the larger Hankel-structured matrices into spatial coefficients and coil basis functions.

(11) $$\|H^{P}\left(x_{s}\right){\|}_{*}=\underset{U_{S},\;V_{S}}{\min}\frac{1}{2}\left(\|U_{S}\|{}_{F}^{2}+\|V_{S}\|{}_{F}^{2}\right)$$

Hence, using the nuclear norm minimization under the matrix factorization constraint, Equation (9) can be rewritten as:

(12) $$E\left(x_{s},U_{S},V_{S}\right)=\underset{x_{s},\;U_{S},V_{S}}{\min}\|\left(H^{P}\left(y\right)-H^{P}\left(x_{s}\right)\right)N_{s}^{c}\|{}_{F}^{2}+\frac{\lambda_{\ell }}{2}\left(\|U_{S}\|{}_{F}^{2}+\|V_{S}\|{}_{F}^{2}\right)$$

subject to $H^{P}\left(x_{s}\right)=U_{S}V_{S}$.

In Equation (11), the object function is nonconvex in terms of the unknowns $x_{s},\;U_{S},$ and $V_{S}$, and thus the global optimal solution is not guaranteed. However, the object function is convex with respect to one variable when the other variables are held constant. To achieve nonconvex optimization, we employ an ADM algorithm to update $x_{s},\;U_{S},$ and $V_{S}$ in an alternating fashion, to iteratively find suboptimal solutions, until the cost function stops decreasing [19,20,21].

Minimization with respect to $x_{s}$: The cost function in Equation (12) is reduced to:

(13) $$E\left(x_{s}\right)=\underset{x_{s}}{\min}\left\|(H^{P}\left(y\right)-H^{P}\left(x_{s}\right)\right)N_{s}^{c}\|{}_{F}^{2}+\frac{\alpha }{2}\|U_{S}V_{S}-H^{P}\left(x_{s}\right){\|}_{F}^{2}$$

where α is a penalty parameter that balances slice separation and signal recovery.

Minimization with respect to $U_{S}$: The cost function in Equation (12) is reduced to:

(14) $$E\left(U_{S}\right)=\underset{U_{S}}{\min}\frac{\lambda_{\ell }}{2}\|U_{S}\|{}_{F}^{2}+\frac{\alpha }{2}\|U_{S}V_{S}-H^{P}\left(x_{s}\right){\|}_{F}^{2}$$

Minimization with respect to $V_{S}$: The cost function in Equation (11) is reduced to:

(15) $$E\left(V_{S}\right)=\underset{V_{S}}{\min}\frac{\lambda_{\ell }}{2}\|V_{S}\|{}_{F}^{2}+\frac{\alpha }{2}\|U_{S}V_{S}-H^{P}\left(x_{s}\right){\|}_{F}^{2}$$

Each subproblem in Equations (13)–(15) is convex, and a global optimal solution can thus be found for each subproblem. The quadratic subproblems are all solved in an alternating fashion using the nonlinear conjugate gradient (CG) algorithm.

### 2.4. Experimental Setup

Experimental studies were performed on a 3T PRISMA scanner (Siemens Healthineers, Erlangen, Germany) equipped with a 20-channel head coil using an inversion recovery (IR) gradient echo (GE) EPI sequence. All experimental procedures were performed under the approval of the Institutional Review Board at the Sungkyunkwan University (Suwon, Korea). An informed written consent was obtained from each volunteer prior to imaging. Fully sampled brain data were acquired with the following imaging parameters: TR/TE = 3 s/23 ms, FOV = 240 × 240, matrix size = 120 × 120, slice thickness = 2 mm, number of slices = 25, and 15 IR scans (inversion times TI = 50, 250, 450, 650, 850, 1050, 1250, 1450, 1650, 1850, 2050, 2250, 2450, 2650, and 2850 ms). All SMS image reconstructions were performed offline on a personal computer with 2.3 GHz CPU and 32 GB RAM using MATLAB (Mathworks Inc., Natick, MA, USA). For faster reconstruction, the acquired data sets were reduced to 12 channels using standard SVD coil compression techniques [22,23]. Prior to data acquisition, a FOV-matched low resolution 2D GRE image was also acquired with 32 fully sampled phase encoding lines at the center of *k*-space for calibration. To retrospectively emulate SMS data acquisition, slice-specific CAIPI-induced phase was added to each slice in *k*-space, before combining the slices into an overlapped slice. For reconstruction, a kernel size of 5×5 (phase encoding × readout) was used in the Hankel-structured matrix, and the low-rank property was imposed on the Hankel block matrix with a total number of 120 spatial coefficients and coil basis, respectively. The cut-off singular value was set toe 0.05 max for the null space selection. The performance of the proposed method depends on the choice of the regularization parameters $\lambda_{\ell }$ and α. Here, we manually set $\left(\lambda_{\ell },\alpha \right)$ to small values (1.0e−7, 2.0e−6) to minimize slice leakage artifacts without perturbing measured SMS data. For visual evaluations of image quality, error maps were produced by calculating the difference between the reference and estimated images. Quantitative comparison was also provided in terms of the normalized root mean square error (nRMSE): $\frac{1}{\max\left(I_{s}\right)}\sqrt{\sum_{s=1}^{N}\frac{1}{N}{\left(I_{s}-{\hat{I}}_{s}\right)}^{2}}$, where $I_{s}$ and ${\hat{I}}_{s}$ are reference and estimated images, respectively. N is the total number of pixels. To demonstrate the benefits of imposing multiparametric null space and low rank, we also performed kernel-based and null space-based SMS reconstructions (i.e., SP-SG and SMS-HSL). The null space rank order of SMS-HSL was the same as that of the proposed method. For SP-SG, kernel estimation was regularized with Tikhonov penalty, and kernel sizes and regularization parameter were selected by visual inspection.

(16) $$SI=SI_{0}\left[1-2e\left(\frac{TI}{T1}\right)+e\left(-\frac{TR}{T1}\right)\right]$$

The term including TR could be negligible (TR > 5 × T1). However, it needs a long scan time because the T1 values at 3T are around 1000–2000 ms. Thus, in this work, we employed the above signal model including TR as a fitting model to estimate the exact T1 values of brain tissues. After reconstruction, T1 map were estimated using pixelwise nonlinear least square fitting.

## 3. Results

**Figure 3:**
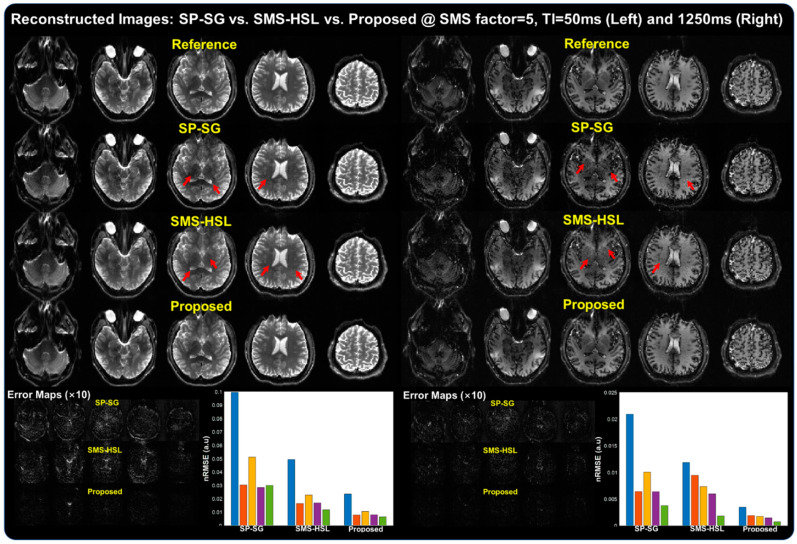
Images, error maps, and nRMSEs generated using SP-SG, SMS-HSL, and our proposed method at an SMS factor of 5 according to different contrasts (TIs = 50 ms and 1250 ms). SP-SG suffers from severe noise (third slice) and aliasing (fourth slice) due to the ill-conditioning of the inverse problem while SMS-HSL mitigates the artifacts with generalized null space kernels. Finally, our proposed method gives the best reconstruction results, owing to the lower rank of the Hankel-structured matrix. The visual quality looks similar for different Tis (Figure S1).

Figure 3 shows reconstructed images, error maps, and nRMSEs for SP-SG, SMS-HSL, and the proposed method at an SMS factor of 5 for two of the TIs (50 and 1250 ms) out of a total of 15 IR scans. The SP-SG reconstructed images exhibit significant noise amplification in the third image and some structured aliasing artifacts in the fourth image with TI = 50 ms and similarly with TI = 1250 ms (red arrows), in the middle of the FOV. The visual quality of the SP-SG breaks down at longer TIs due to the low SNR in signal nulling. The SMS-HSL case does not display strong artifacts, although close inspection does reveal that some noise is still present. This is to be expected due to the improved conditioning of the matrix inversion through generalized null space kernels, which are all independent of each other. Notably, our proposed method consistently reconstructs high-quality images that are devoid of visible artifacts for two of the TIs, attributed to the increased low rank of the Hankel-structured matrices of multi-TI data. Consistent with the results shown in the reconstructed images, the corresponding error maps tend to exaggerate contrast contamination with noises particularly in SP-SG while yielding the lowest nRMSEs in the proposed method compared to SP-SG and SMS-HSL.

**Figure 4:**
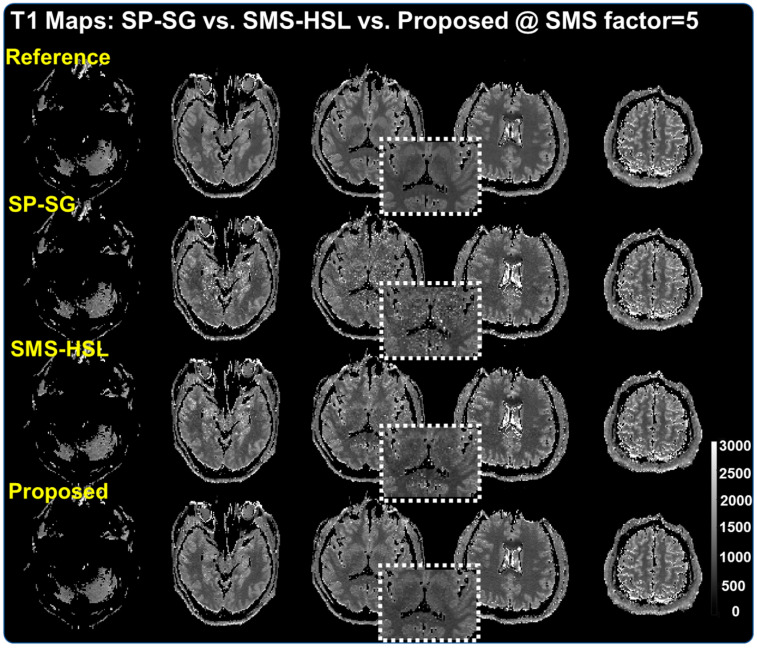
Corresponding T1 maps calculated from SP-SG, SMS-HSL, and our proposed method at an SMS factor of 5. The results from SP-SG exhibit uneven T1 values in the same tissue due to the presence of severe noise while SMS-HSL results display improved conditioning with generalized null space kernels although some noise is still visible. The proposed method provides the best T1 values, with high precision, due to the improved low rank of the Hankel structured matrix.

Figure 4 compares the corresponding T1 maps (Figure S2) with enlarged figure in the white dotted boxes, calculated from the reconstructed images of multi-TI data sets at an SMS factor of 5. In line with the reconstruction results, the T1 values that are estimated from SP-SG deviate from the correct values, even for the same tissue, due to the severely amplified noise. In contrast, SMS-HSL provides dramatic improvement over SP-SG despite the presence of some noise. Finally, with the proposed method, the T1 maps have better SNR and the T1 values have higher precision than those of SP-SG and SMS-HSL, consistent with the reconstruction results.

**Figure 5:**
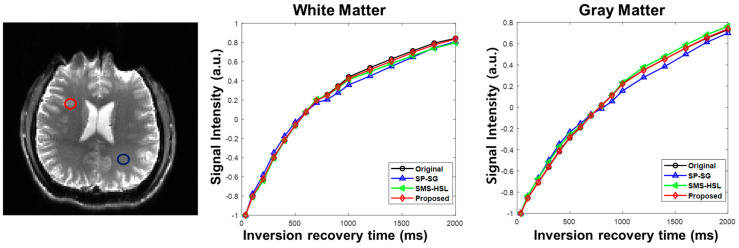
T1 signal intensity curves of white and gray matter regions. The curves are generated from the reference and the reconstructed images by averaging the voxels in each region of interest. The signal curves from the proposed method strictly follow the decay curves of the reference, SP-SG and SMS-HSL.

For quantitative evaluation, T1 relaxation curves are shown in Figure 5. The values within manually chosen 3 × 3 pixel white and gray matter regions of interest (ROI) were averaged. The T1 values for white and gray matter follow the pattern of the aforementioned results. The proposed method follows the true signal intensity curve much more accurately than the other methods, which display some signal bias due to signal nulling during inversion recovery. The disparity between the proposed method and the other methods is more pronounced for white matter.

## 4. Discussion

In this work, we extend SMS-HSL to the context of dynamic MR imaging. This is achieved by concatenating the SMS-HSL matrices of different contrasts to yield a larger, more redundant low-rank dynamic imaging data matrix. This new reconstruction model utilizes the null-space-based slice unfolding followed by enforcing the low-rankness of the block Hankel matrix independently for each slice. The improved low rank is proof of the existence of null space filters in the k-space domain whereby k-space data are represented in a lower dimensional signal subspace, enabling the unfolding of each slice. Following up on our previous work on SMS reconstruction [17], we developed a new algorithm to incorporate the null and signal subspaces into a single optimization problem under a joint framework. We demonstrate the superior performance of the proposed algorithm in the accelerated recovery of SMS MR parameter data, using in vivo MR brain scans.

T1 mapping commonly relies on the inversion recovery sequence where EPI acquisitions are continuously applied during signal recovery followed by a computation of the T1 values during postprocessing. Multishot EPI acquisitions potentially yield motion mismatches and shot-to-shot phase variations between scans. These issues could be addressed by adopting SMS acceleration, which brings down the time to enable a single-shot SMS acquisition. The advanced technique presented in this work provides a drastic improvement in image quality at high SMS accelerations, as observed in Figure 3, while helping mitigate some of the involuntary motion and phase variation issues.

In terms of computation, the SVD-free reconstruction algorithm (LMaFit), which is based on matrix factorization, was employed. The proposed algorithm is still computationally demanding due to the existence of a parameter dimension. To mitigate this computational issue, the matrix inversion of the proposed method in Equation (7) can be precomputed. In addition, the proposed algorithm can be efficiently parallelized using a combination of multicore CPUs with GPUs, such that the null space estimation, which requires high computational cost, is performed on multicore CPUs for each slice while the matrix multiplication for data synthesis is implemented on GPUs with a large number of cores.

Our proposed method is not restricted to T1 quantification. It can be easily applied to the mapping of different MR parameters such as T2, T2 *, and diffusion with acquisitions based not only on single-shot EPI sequences but also on fast spin echo (FSE) or gradient echo (GRE) sequences.

## 5. Conclusions

In conclusion, we developed and evaluated a dynamic version of SMS-HSL in the reconstruction of SMS imaging data by simultaneously imposing noise and signal subspaces to the calculation of T1 maps. The proposed SMS-HSL method was demonstrated to have substantial advantages over competing methods in providing high quality reconstructions and high precision T1 maps with up to fivefold SMS acceleration.

## Data Availability

Additional data are available from the first/corresponding author upon reasonable request.

## Supplementary Materials

The proposed method was additionally evaluated on human brain from different data set of the healthy subject available online at https://www.mdpi.com/article/10.3390/tomography7040047/s1, Figure S1: reconstructed images using SP-SG, SMS-HSL and proposed method, Figure S2: the corresponding T1 maps.
