# Supplementary material for: Simultaneous Multislice Brain MRI T1 Mapping with Improved Low-Rank Modeling
Source: Tomography. 2021 Oct 7;7(4):545–54. doi: 10.3390/tomography7040047 (PMC8544713; doi:10.3390/tomography7040047)
Supplement: Supplementary file 1 [file tomography-07-00047-s001.zip › tomography-1360711-supplementary.pdf]

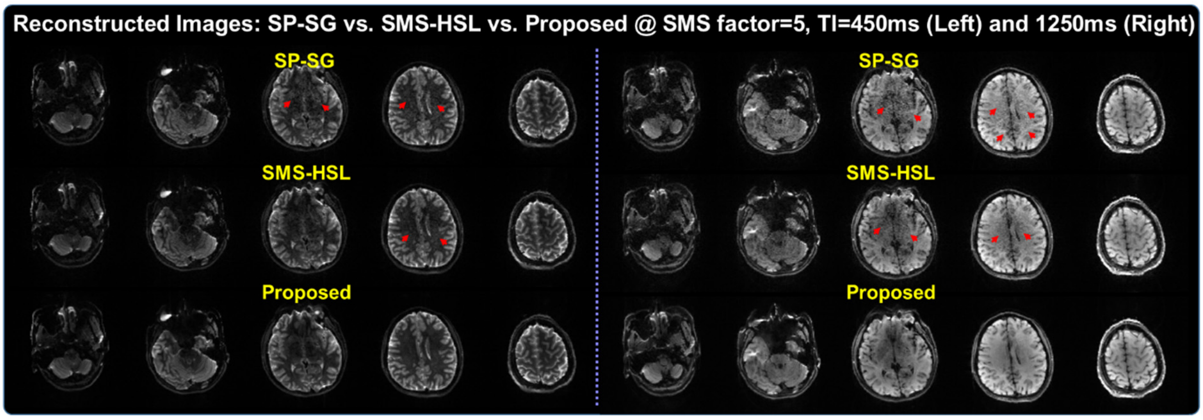

Figure S1. Reconstructed images using SP-SG, SMS-HSL, and proposed method at an SMS factor of 5 according to different contrast (TI = 450ms, and 1250ms). SP-SG has noise amplification (3rd slice) and aliasing (4th slice), especially in the middle of the brain (red arrows). The SMS-HSL yields a minor improvement in terms of noise amplification (red arrows). On the other hand, proposed method provide the best reconstruction results owing to the lower rank of the Hankel structed matrix. The visual quality looks similar for different TIs.

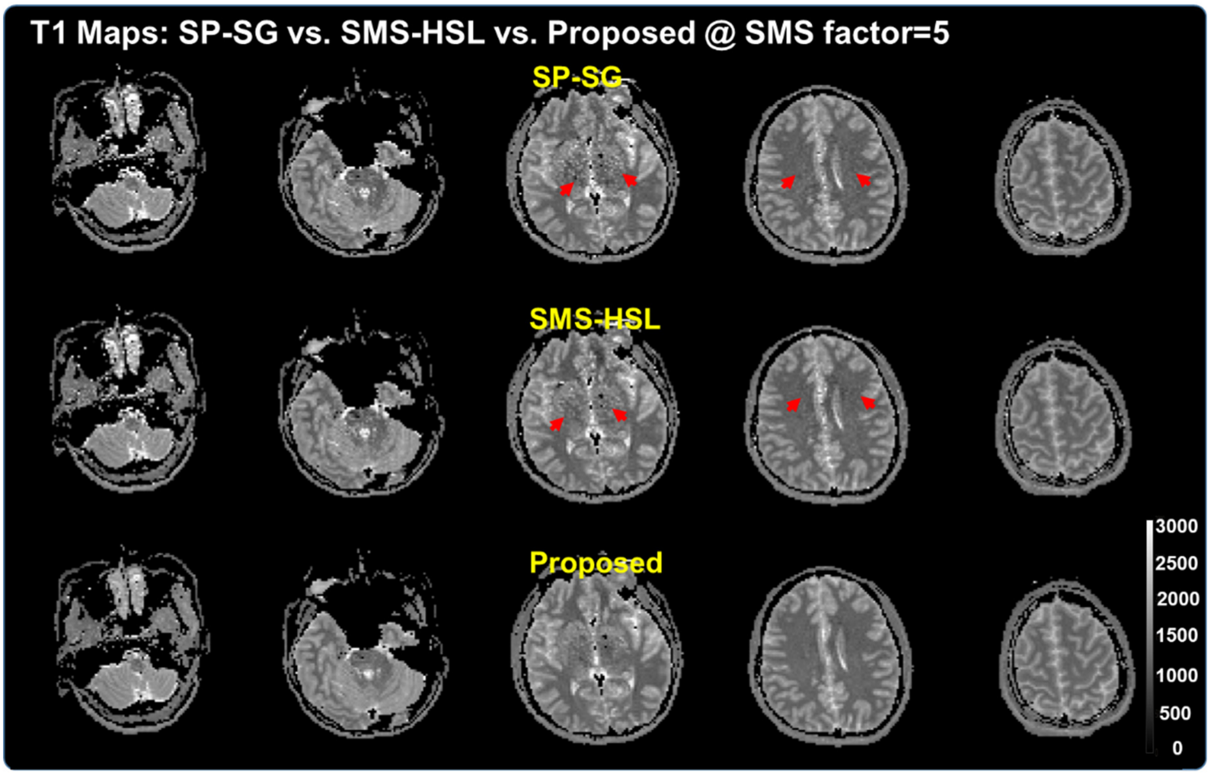

Figure S2. T1 maps calculated from SP-SG, SMS-HSL, and the proposed method at and SMS factor of 5. Note that the results from proposed method are the most consistent T1 values, whereas T1 maps from SP-SG and SMS-HSL are severely amplification of the noises in the middle of images (red arrows).
